# Physical Characteristics of Durum Wheat Dough and Pasta with Different Carrot Pomace Varieties

**DOI:** 10.3390/gels11070481

**Published:** 2025-06-22

**Authors:** Marian Ilie Luca, Mădălina Ungureanu-Iuga, Ana Batariuc, Silvia Mironeasa

**Affiliations:** 1 Faculty of Food Engineering, “Ştefan cel Mare” University of Suceava, 13 Universitatii Street, 720229 Suceava, Romania; marian.luca@usm.ro (M.I.L.); silviam@fia.usv.ro (S.M.); 2 Integrated Center for Research, Development and Innovation in Advanced Materials, Nanotechnologies, and Distributed Systems for Fabrication and Control (MANSiD), “Ştefan cel Mare” University of Suceava, 13th Universitatii Street, 720229 Suceava, Romania; 3 Sanitary, Veterinary and Food Safety Directorate of Suceava, 2nd, Scurta Street, 720223 Suceava, Romania; ana.batariuc@usm.ro

**Keywords:** texture, rheology, waste valorization, functional foods, carrot by-product, gel viso-elasticity

## Abstract

Carrot pomace is a valuable, underutilized by-product suitable for obtaining novel foods. The durum wheat dough and pasta network structure is affected by fiber-rich ingredients like carrot pomace, leading to changes in rheological and texture parameters. In this context, this paper aimed to evaluate the rheological, textural, and color properties of durum wheat dough and pasta as affected by different varieties and addition levels of carrot pomace. For this purpose, oscillatory dynamic rheological tests, compression mechanical texture evaluation, cooking behavior observation, and reflectance color measurements were made. The results indicated that carrot pomace has a strengthening effect on the durum wheat dough protein–starch matrix, while the maximum creep compliance decreased with the addition level increase. A delay in starch gelatinization was suggested by the evolution of visco-elastic moduli during heating. Dough hardness and gumminess increased (from 2849.74 for the control to 5080.67 g for 12% Baltimore, and from 1073.73 for the control to 1863.02 g for 12% Niagara, respectively), while springiness and resilience exhibited a reduction trend (from 100.11% for the control to 99.50% for 12% Sirkana, and from 1.23 for the 3% Niagara to 0.87 for 12% Belgrado respectively) as the amount of carrot pomace raised. An increasing tendency of pasta solids loss during cooking and fracturability was observed with carrot pomace addition level increase. Color properties changed significantly depending on carrot pomace variety and addition level, indicating a reduction in lightness from 71.71 for the control to 63.12 for 12% Niagara and intensification of red nuance (0.05 for the control vs. 2.85 for 12% Sirkana). Cooked pasta elasticity, chewiness, gumminess, hardness, and resilience increased, while adhesiveness and stickiness decreased as the level of carrot pomace was higher. These results can represent a starting point for further industrial development of pasta enriched with fiber-rich ingredients like carrot pomace. The study highlights the possibility of using a fiber-rich waste stream (carrot pomace) in a staple product like pasta, providing a basis for clean-label pasta formulations. In addition, the novelty of the study consists in highlighting how compositional differences of different carrot pomace varieties lead to distinct effects on dough rheology, texture, color, and cooking behavior.

## 1. Introduction

In recent years, various studies have explored the use of by-products of other food-processing products as ingredients to create innovative nutritionally enriched pasta with increased functional value [1,2,3]. When wheat flour is substituted with vegetable by-products, different products with enhanced nutritional, functional, and organoleptic features can be obtained, but they play a relevant technological role in dough development and processing.

The studies reported changes in the behavior of wheat dough upon these ingredients as fiber and bioactive compound sources. From a rheological point of view, developed wheat dough is a viscoelastic gel whose parameters are frequency dependent [4]. Being a complex system, dough can be characterized in a fundamental and quantitative manner by investigating its viscoelastic properties [5,6]. Additionally, useful information on dough quality and product manufacture is offered by the rheological properties studied in the linear viscoelastic region, which are related to the structural and compositional features of the material [7,8]. In addition, the dough textural characteristics and pasta textural, physical, and color features are affected by the compositions of the various by-products added in pasta formulation [7,9].

A pasta enriched with carrot pomace resulting from juice extraction could represent a healthy choice for consumers because this ingredient is remarked for its high content of total carotenoids, influenced by processing and extraction methods [10], as well as in fibers (37–48%), reducing sugars (8–9%), minerals (5–6%), etc. [11]. The chemical composition of carrot pomace is also influenced by the carrot variety and drying method used for preservation [12,13]. Incorporation of carrot pomace in wheat flour as a fiber source associated with bioactive compounds induces considerable changes in gel viscoelasticity during wheat dough development, depending on the kind of fiber. It has been reported in various studies that insoluble fiber increased the viscoelastic moduli and, in contrast, soluble fiber decreased the gel strength.

The viscoelastic parameters are also affected by the fiber quantity, the ratio between soluble and insoluble fiber, particle size, porosity, specific surface area, and others [14]. Increasing viscosity and affinity towards gel formation is related to soluble dietary fiber. The fiber can have a direct effect on the viscoelastic gel, contributing to dough elasticity, or an indirect effect related to variation in water absorption. The decrease in water content determined a rise of dynamic moduli G′ and G″ [15]. The soluble fibers incorporated in certain doses in wheat flour strengthen the structure of the dough; meanwhile, higher amounts of insoluble fibers have an adverse effect on the formation of the gluten network because of gluten–fiber interaction. Moreover, insoluble fiber induces the disruption of the viscoelastic system, resulting in weaker dough due to the fiber–starch competition for water, influencing pasting and gelling, whereas soluble fiber increases dough stability due to its integration into the dough system [16]. Therefore, the insoluble fibers, disrupting the gluten network, lead to changes in dough extensibility and elasticity, causing lower quality of products. A similar finding was reported for the bread quality [17].

In addition to the application of oscillatory stress to determine the dynamic moduli, the viscoelastic properties of fiber-enriched dough can also be defined by applying constant stress over a period of time, conducted through the creep and recovery test [14]. Compliance (J) describes the elastic or viscous deformations that occur in dough at a constant stress. 

The detrimental effects of various fibers on texture were reported in different studies and depend greatly on fiber properties, but inconsistent effects were frequently found [18]. The addition of carrot pomace to pasta products can affect texture parameters, such as firmness and stickiness of cooked products, material release into cooking water, and color. Many studies reported the impact of carrot pomace incorporation in pasta and baked goods (Table 1). To our knowledge, this is the first research regarding the evaluation of carrot pomace variety on durum wheat pasta quality since none of the existing papers mention a comparison between different varieties.

In this context, this work aimed to investigate the impact of different addition levels (0–12%) of carrot pomace from four varieties (Baltimore, Belgrado, Niagara, and Sirkana) on durum wheat pasta dough rheological and textural properties, as well as on dry and cooked pasta texture and color. The hypothesis was that carrot pomace addition modifies pasta dough rheological behavior and has a significant impact on pasta texture and color parameters. These results could be helpful for further development of novel functional products.

## 2. Results and Discussion

### 2.1. Dough Texture Properties

**Figure 1:**
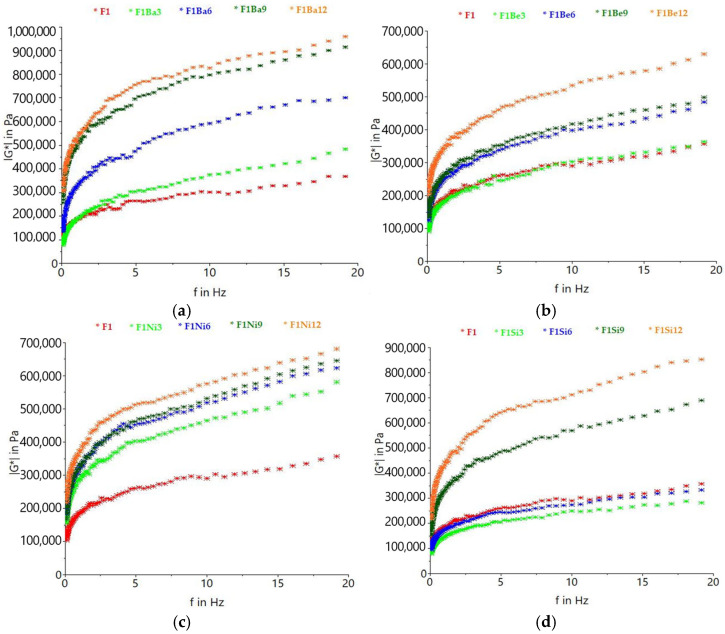
Variation in complex modulus with frequency for durum wheat (F1) dough with different carrot pomace varieties: Baltimore (Ba) (**a**), Belgrado (Be) (**b**), Niagara (Ni) (**c**), and Sirkana (Si) (**d**), and addition levels (3, 6, 9, and 12%).

Complex modulus (G*) indicates dough strength and increases as the addition level of carrot pomace was higher for all the varieties studied (Figure 1). Compared to the control, the samples containing Baltimore, Belgrado, and Niagara pomace exhibited higher G* values, while dough samples with 3 and 6% Sirkana pomace showed lower G*. 

Hydration has an essential role in changing protein structure in dough, while the physical and chemical interactions between protein molecules affect dough rheology [26]. As carrot pomace is rich in fiber [12], the hydration properties of durum wheat flour changed with the addition level, leading to different G* values. Two mechanisms can explain the increase in G* with carrot pomace addition level: either there is a diminishing of lubrication by water as a result of the competition for water between gluten and carrot fiber, or the fibers play the role of a filler in the dough matrix [27]. Other studies reported the increase in common wheat G* as the amount of fiber-rich ingredients like grape seeds or barley soluble fibers increased [28,29]. The results obtained suggest that carrot pomace addition determined poorly extensible or more deformation-resistant dough, as indicated by the increase in G*. The incorporation of carrot pomace, which is rich in fibers, into wheat dough increases its water absorption capacity and interferes with the proper formation of the gluten network [30]. Alterations in the viscoelastic behavior of dough, particularly when supplemented with insoluble dietary fiber, are due to disruptions in gluten structure, competitive interactions with starch, and the high water-binding capacity of fiber [31]. Fibers, proteins, and sugars from carrot pomace can form hydrogen bonds with water and/or starch and gluten protein molecules, leading to a decrease in the amount of free water and overall mobility of the system, which determines higher dough firmness, viscosity, and consistency [32]. The presence of phenolic compounds from carrot pomace could also explain the changes in G*. Phenolic compounds from carrot pomace can interact with proteins and/or polysaccharides by complexation. This reaction can occur “via hydrogen bonding between hydroxyl groups of phenols and the carbonyl group of peptide rest of proteins” [8]. The compounds formed can be stabilized through covalent bonds and ionic bonds between phenolate and anion or cationic sites of protein molecules [8]. Another way of interaction between proteins and polyphenols is the hydrophobic interactions, which suppose that a “polyphenol molecule attaches onto the protein surface, or cross-links with different protein molecules” [8]. Similarly, there are complexation reactions between polyphenols and polysaccharides, with the mention that the hydrophobic interactions are promoted by hydrophobic cavities [8]. 

**Figure 2:**
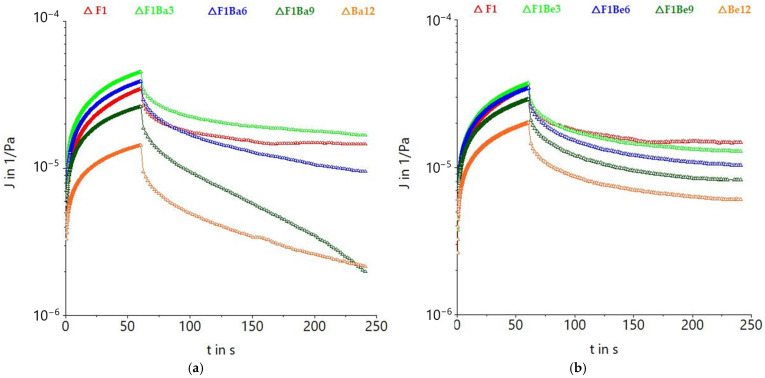

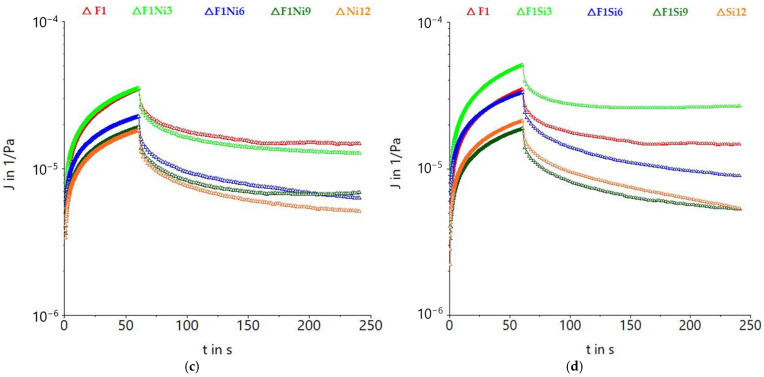
Creep and recovery curves for durum wheat (F1) dough with different carrot pomace varieties: Baltimore (Ba) (**a**), Belgrado (Be) (**b**), Niagara (Ni) (**c**), and Sirkana (Si) (**d**), and addition levels (3, 6, 9, and 12%).

Creep and recovery curves are displayed in Figure 2. Generally, the addition of carrot pomace resulted in a decrease in maximum creep compliance (J_max_), so a strengthening effect was observed. This strengthening effect suggested that cross-linking between pomace compounds and wheat proteins took place and that smaller amounts of free water were available in the dough system [33]. 

It has been suggested that the strengthening of dough with fiber-rich ingredients may be due to conformational alterations of gluten proteins resulting from the addition of fibers, causing a stiffer dough matrix [34]. Furthermore, rheological behavior modifications can also be due to the microstructural reordering caused by the presence of fiber particles from carrot pomace since these particles are distributed within the starch–protein network [34]. Dough recovery also decreased as the addition level of carrot pomace increased, with some exceptions. It has been proved that polyphenols from plants impact dough rheology [6] and may exert a strengthening effect [7]. Similar to our findings, Lei et al. [35] also reported that the increase in wheat bran insoluble fiber content in noodle dough resulted in a decrease in creep deformation of the samples in both the creeping step and the recovery step due to the restriction of the hydration of starch and protein in pasta dough, leading to higher mechanical strength.

**Figure 3:**
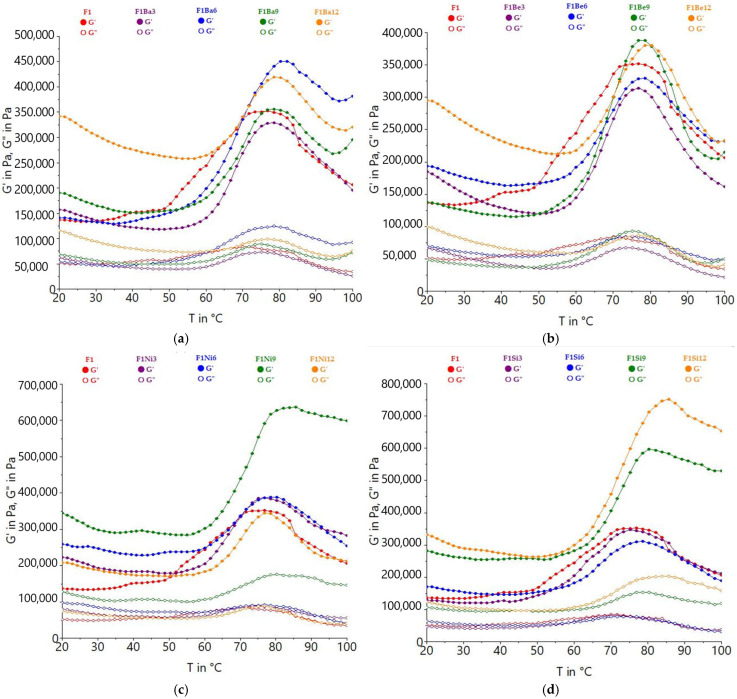
Variation in storage (G’) and loss modulus (G’) with temperature for durum wheat (F1) dough with different carrot pomace varieties: Baltimore (Ba) (**a**), Belgrado (Be) (**b**), Niagara (Ni) (**c**), and Sirkana (Si) (**d**), and addition levels (3, 6, 9, and 12%).

Temperature sweep test curves indicated three stages of dough behavior during heating (Figure 3): in the first one, the decrease in G‘ and G” occurs until a certain temperature, then the visco-elastic moduli increase greatly until the maximum gelatinization temperature is reached, then they decrease. An increasing trend of visco-elastic moduli and gelatinization temperatures as the addition level was higher was observed, with some exceptions. A delay of gelatinization was observed as the amount of carrot pomace increased. Hao et al. [36] also reported higher gelatinization temperatures when alfalfa powders were added to the wheat dough, which could be related to the presence of fibers, which decreased the ability of water to be effectively involved in starch grain swelling and, therefore, delayed gelatinization. The decrease in visco-elastic moduli up to the initial gelatinization temperature could be due to a kinetic effect determined by the temperature increase that may exert a weakening of the structure [37]. Similar to our findings, Ahmed et al. [38] observed a strengthening effect of fibers on the wheat dough during non-isothermal heating without major changes in gelatinization temperatures and compared this effect with nanoclay addition to biopolymer/bio-nanocomposite, where almost all Tg values were unchanged and the mechanical strength increased [38,39]. After the maximum G’ was reached, increasing heating temperature allowed the energy to enter the inside of the starch granule to break down the rest of the crystalline structure, and on further heating, G’ was reduced [40]. This decrease at higher temperatures can be explained by the softening of the granules, the rise of their deformability, and the break and leaching of granule amylase [38]. Carrot pomace incorporation increased gelatinization temperatures by restricting water access to the amorphous areas of the starch granules due to its higher content of fibers [34]. Moreover, hydrophilic phenolic compounds can engage in non-covalent interactions with starch molecules while simultaneously competing for available water. These interactions may modulate the physicochemical environment, including pH, thereby affecting starch granule hydration, altering gelatinization kinetics, and influencing pasting behavior [41].

Dough texture parameters are presented in Table 2. Hardness and gumminess showed an increasing trend as the addition level of carrot pomace was higher. On the other hand, springiness and resilience were reduced by the increase in carrot pomace amount.

Cohesiveness did not show any significant differences among samples, although a slight decrease was found. Asadi et al. [42] also reported an increase in dough gumminess and a reduction in springiness and cohesiveness as the addition level of carrot pomace was higher. Fiber from carrot pomace can fragment the gluten matrix, hold off gluten hydration, and compete for water with starch and proteins in the dough system [42,43], which may cause hardness and gumminess to rise. The gluten dilution effect could also have contributed to dough hardening, along with the interactions between fibers and gluten proteins [44]. The reduction in cohesiveness can be due to the presence of carrot pomace particles in the system, which may have disrupted the structure [42]. A previous study reported an increase in dough hardness when bilberry and blackcurrant pomace were added to dough [45] as a result of the ability of pomaces to bind water since dough hardness is determined by the moisture content and the ability of components to bind water. Chetrariu and Dabija [46] observed an increase in dough hardness when spent grain from malt whisky was incorporated into spelt pasta formulation.

### 2.2. Pasta Characteristics

**Figure 4:**
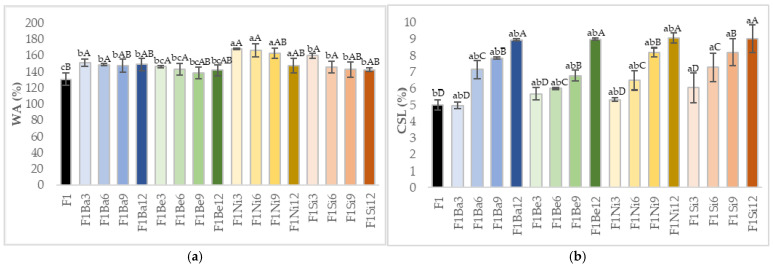
Cooking behavior of pasta: (**a**) water absorption (WA); (**b**) cooking solids loss (CSL); a–c—different small letters suggest significant differences (*p* < 0.05) among carrot pomace varieties; A–D—different uppercase letters suggest significant differences (*p* < 0.05) among addition levels; Ba—Baltimore, Be—Belgrado, Ni—Niagara, Si—Sirkana, F—durum wheat, 3–12—addition levels.

Pasta cooking characteristics are important quality traits. Water absorption during cooking decreased (Figure 4) with the increase in carrot pomace concentration for all the varieties studied, with the lowest values being observed for the control. The decrease in WA could be related to the presence of polyphenols in carrot pomace, which affected the conformation of gluten proteins and the starch granules structure with a possible impact on pasta water absorption; a similar observation was made for pasta with elderberry juice [47]. As expected, cooking solids loss increased as the addition level increased, with the smallest changes being observed for 3% pomace of all carrot varieties (Figure 4).

Uncooked pasta is formed by a continuous protein network that entraps starch grains. The drying process of pasta can negatively affect its quality by denaturing gluten proteins, which are crucial for maintaining the desired protein cross-linking [48]. When pasta is cooked, its matrix readily absorbs water, causing starch granules to swell and lose their rigid structure. This swelling releases soluble carbohydrates, such as amylose, from the starch into the cooking water, explaining the observed solid losses [47]. Crucially, though, most gluten proteins stay intact, acting to bind the starch granules and thus limit the amount of solid material lost during cooking [47,49]. Thus, the compactness of the pasta network significantly influences how much water the starch granules can absorb during the cooking process. Studies revealed that augmenting the fiber content in the dough matrix breaks the starch–protein matrix, leading to higher CSL [50]. Our results are in agreement with a previous study regarding the effect of soybean and carrot pomace addition on durum wheat pasta quality [51], which revealed higher cooking loss as the addition level increased.

**Figure 5:**
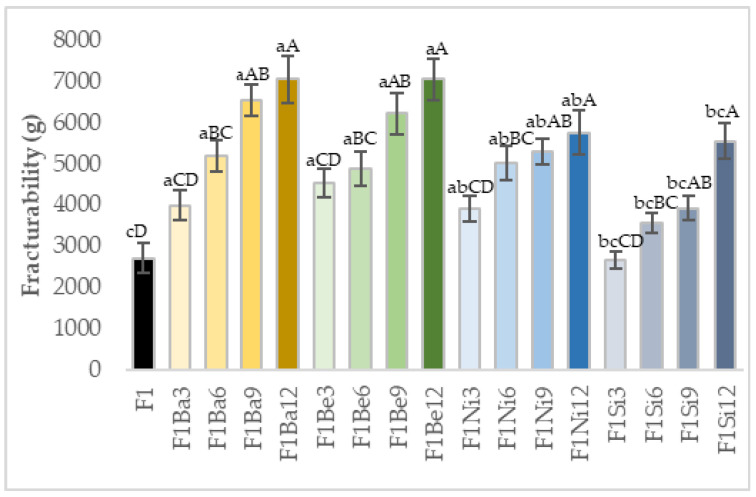
Fracturability of uncooked pasta: a–c—different small letters suggest significant differences (*p* < 0.05) among carrot pomace varieties; A–D—different uppercase letters suggest significant differences (*p* < 0.05) among addition levels, Ba—Baltimore, Be—Belgrado, Ni—Niagara, Si—Sirkana, F—durum wheat, 3–12—addition levels.

Fracturability is a measure of pasta’s resistance to breakage, and it is important for transport and package choice. An increase in the pasta’s maximum force to break (fracturability) was obtained as the addition level of carrot pomace was higher for all the varieties considered (Figure 5). The lowest fracturability values were reported for control and F1Si3. Lou et al. [52] also observed an increase in biscuits’ fracturability when grape pomace powder was incorporated and associated this behavior possibly to the linkage between polyphenols and protein or starch, causing dough strengthening. Another study demonstrated that the addition of garlic powder or grits up to 1% increased pasta fracturability as a result of pasta structure strengthening by soluble fibers [53]. Ismail and Aziz [54] reported that the rise of pumpkin puree concentration led to a higher force required to give the first crack of the stick snacks.

Carrot pomace addition resulted in smaller lightness of pasta and an intensification of red nuance (increased in a* values) depending on the carrot variety used (Table 3). Furthermore, the yellow nuance (b* values) decreased as the addition level increased. The white index (WI) also exhibited a decreasing trend, while the yellow and browning index exhibited irregular variations with the addition level. Carrot pomace varieties have different color properties depending on the chemical composition, and in orange varieties, the color is related to the concentration and type of carotenoids [12,55]. 

Our previous study indicated that the Sirkana variety exhibited the highest beta carotene concentration and yellow index [56], which can explain the higher pasta red and yellow nuance compared to the other samples. The yellow color of pasta can be associated with good-quality products and determine higher consumer acceptability [57]. Thus, the addition of carrot pomace could lead to better acceptance of the product due to its yellowish color. Carrot flour was proven to have a more pronounced effect on b* compared to commercial β-carotene additives in pasta [57]. Similar to our observations, Prerana and Anupama [58] reported lower L* values as the quantity of carrot puree increased in fresh noodles, while the red nuance was accentuated. Carrot pomace color depends on the variety, and for orange carrots, it is due to the presence of carotenoids such as carotenes and xanthophylls [11,55]. Usually, orange carrots present significant content of β-carotene and α-carotene but also contain lutein and zeaxanthin, while red carrots also have lycopene besides the amount of α-/β-carotene and lutein [59]. Carotenoids are sensitive to temperature, light, and oxygen, and during pasta drying, their isomerization and oxidative reactions can result in thermal degradation, thereby diminishing the color attributes, sensory properties, and nutritional value of the product [60].

Cooked pasta texture parameters are presented in Table 4. Elasticity, cohesiveness, hardness, adhesiveness, and stickiness increased with carrot pomace level increase, with some exceptions. 

Chewiness, gumminess, and resilience exhibited a reduction as the amount of carrot pomace was higher. These variations were influenced by the carrot pomace variety. Kultys et al. [19] also reported lower adhesiveness and chewiness for pasta with carrot pomace. Adhesiveness indicates the force necessary to remove the sampler from the pasta sample, and it depends on the quantity of amylose washed out of the gelled starch granules [61]. Xu et al. [62] reported lower chewiness and gumminess values of pasta when 15% apple pomace was incorporated. The inclusion of carrot pomace in starch-based formulations offers a dual benefit: enhancing dietary fiber and influencing the overall microstructure. By analogy, as observed by Rocha-Parra et al. [63] for apple pomace, carrot pomace’s superior water absorption capabilities could lead to delayed starch gelatinization and alter pasting properties. The authors also affirmed that apple pomace affects pasta microstructure. It is reasonable to infer that those comparable microstructural alterations occurred in the carrot pomace-enriched pasta of this study, contributing to their observed textural modifications [63,64]. Similar to our results, Gałkowska et al. [64] observed that the samples containing a higher amount of blackcurrant pomace exhibited higher values of hardness and elasticity, suggesting that the absorption of water by the pasta during boiling dominated the impact of the type of pomace component on pasta hardness. The higher hardness values are desirable in terms of cooking the pasta products in an al dente way. An important role in pasta hardness increase could be attributed to the presence of phenolic compounds in carrot pomace [65]. The samples with higher levels of carrot pomace absorbed less water (Figure 4) compared to those with lower carrot pomace content; therefore, their internal structure was less loosened and consequently more resistant to stretching, as supported by Gałkowska [64].

### 2.3. Correlations and Principal Component Analysis (PCA)

**Figure 6:**
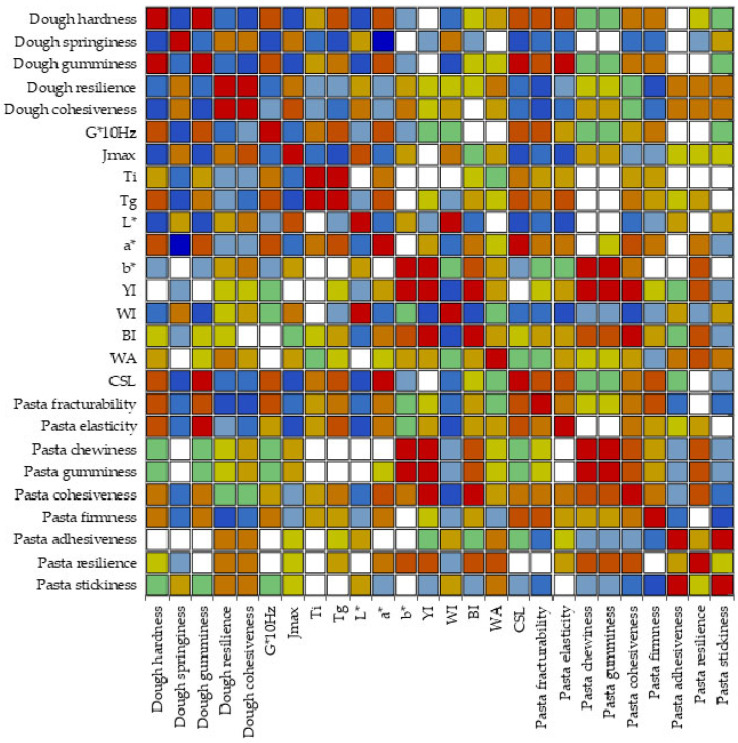
Heatmap for Pearson correlations.

Some correlations were observed between variables, and they are displayed in Table S1 and in Figure 6. Dough texture and rheological parameters were significantly correlated (*p* < 0.05); Ti correlated with dough springiness (*p* < 0.05, r = −0.55), a* (*p* < 0.05, r = 0.52), and CSL (*p* < 0.05, r = 0.54); and T_g_ was correlated with dough springiness (*p* < 0.05, r = −0.68) and gumminess (*p* < 0.05, r = 0.58), a* (*p* < 0.05, r = 0.65), CSL (*p* < 0.05, r = 0.64), pasta elasticity (*p* < 0.05, r = 0.65) and fracturability (*p* < 0.05, r = 0.55). 

Edwards et al. [66] stated that small deformation measurements by using dynamic rheological methods are highly correlated with texture firmness measurements, which is consistent with our results since dough hardness was positively correlated with G*_10Hz_ (*p* < 0.05, r = 0.79) and J_max_ (*p* < 0.05, r = −0.75). Yellow index (YI) was positively correlated with b* (*p* < 0.05, r = 0.84). Significant correlations were observed for WI with L* (*p* < 0.05, r = 0.91), a* (*p* < 0.05, r = −0.49), and YI (*p* < 0.05, r = −0.69). Pasta WA was correlated positively with dough resilience (*p* < 0.05, r = 0.57). CSL was significantly correlated (*p* < 0.05) with all the rheological and textural parameters of dough. Pasta fracturability was correlated with dough hardness, gumminess, resilience, cohesiveness, G*_10Hz_, J_max,_ and T_g_ (*p* < 0.05). Pasta elasticity was significantly correlated (*p* < 0.05) with dough texture parameters, except resilience, J_max,_ and T_g_. A strong positive correlation (*p* < 0.05, r = 0.99) was observed between pasta chewiness and gumminess. Pasta resilience was positively correlated with (*p* < 0.05, r > 0.66) chewiness, gumminess, and cohesiveness of pasta, while pasta stickiness was correlated with adhesiveness (*p* < 0.05, r = 0.89).

Five principal components were selected based on their eigenvalues > 1 (Table 5). The first component (PC1) explained 39,19% of data variability, followed by PC2 with 24.28%, PC3 with 13.75%, PC4 with 7.84%, and PC5 with 4.57%. These components explained a cumulative variance of 89.62%.

The contribution of the variables to each component is presented in Table 6. 

PC1 clustered the variables related to dough texture, with the exception of resilience, a* color parameter, CLS, and pasta fracturability and elasticity. PC2 accounted for the grouping of variables associated with color—b*, YI, BI, and pasta texture—chewiness, gumminess, cohesiveness, and resilience. Dough resilience, WA, pasta firmness, adhesiveness, and stickiness were grouped by PC3, while Tg, L*, and WI were clustered by PC4. The last component (PC5) accounted for the grouping of rheological properties of dough, namely G*_10Hz_, J_max_, and T_i_.

The contribution of the samples to each principal component is shown in Table 7. PC1 clustered F1Ni12, F1Si3, and F1Si6 samples, while F1 and F1Be3 were grouped by PC2. PC3 accounted for the grouping of the highest number of samples—F1Be6, F1Be9, F1Be12, F1Ni3, F1Ni6, and F1Ni9. As indicated by their highest contribution, F1Ba3, F1Ba6, F1Ni9, F1Si9, and F1Si12 were clustered by PC4, while F1Ba9 and F1Ba12 were associated with PC5.

## 3. Conclusions

This study highlighted the impact of carrot pomace from different varieties on durum wheat dough and pasta rheological, textural, and color characteristics. It can be mentioned that the carrot pomace variety had a significant impact only on color properties due to the difference in pigment concentration. The addition level exhibited a great impact on dough: strength increased, rheological behavior during creep and recovery showed a decrease in recovery power, and the gelatinization process during heating was delayed by carrot pomace addition. In addition, dough and pasta texture changed significantly as the level of carrot pomace was higher by increasing hardness and decreasing resilience and chewiness. Pasta fracturability and loss of solids during cooking raised with the carrot pomace addition level, while color properties showed an increase in red nuance and reduction in lightness. This study presents a novel comparative assessment of carrot pomace from different varieties and its influence on durum wheat dough and pasta properties. Unlike previous studies that used a single or unspecified pomace source, this research demonstrates that while variety-specific differences primarily affect color parameters due to pigment content, the additional level of pomace significantly alters the rheological, thermal, and textural behavior of the dough and final product quality. In addition, increased carrot pomace levels led to the strengthened dough, delayed starch gelatinization, and reduced recovery power, as well as increased pasta hardness, fracturability, and cooking loss, findings that deepen understanding of fiber–protein–starch interactions in semolina systems. Further researches are needed to evaluate the impact of carrot pomace from different varieties on pasta’s nutritional, functional, and sensory characteristics.

## 4. Materials and Methods

### 4.1. Materials

Four distinct carrot types—Niagara, Belgrado, Sirkana, and Baltimore—were obtained directly from a farmer in Bacău, Romania. Carrot pomace was obtained by extracting the juice using a Bosch MES 3500 juice extractor [12]. This leftover material was then dried in a 60 °C convection oven for a full day. Following drying, the pomace was ground into a fine powder, ensuring the particle size was smaller than 200 μm by sieving. Durum wheat flour was also purchased from a local market. Dough and pasta samples were obtained by mixing this durum wheat flour (labeled F1) with varying amounts—specifically 3%, 6%, 9%, and 12%—of the powdered pomace from each of the four carrot varieties: Niagara (Ni), Belgrado (Be), Sirkana (Si), and Baltimore (Ba).

The chemical composition of the carrot pomace was presented in our previous studies [12,56]. The carrot pomace from the four varieties displayed varying chemical characteristics. For moisture content, Baltimore registered 4.04%, Belgrado 3.78%, Niagara 5.88%, and Sirkana 5.91% (all determined by SR EN ISO 665:2020 [67]). Regarding fat content (measured by SR EN ISO 659:2009 [68]), Baltimore had 1.00%, Belgrado 1.01%, Niagara 0.70%, and Sirkana 1.13%. Protein levels (analyzed using SR EN ISO 20483:2014 [69]) were 6.87% for Baltimore, 8.01% for Belgrado, 8.84% for Niagara, and 9.14% for Sirkana. The ash content (according to SR EN ISO 2171:2023 [70]) was 5.29% for Baltimore, 5.89% for Belgrado, 5.56% for Niagara, and 5.89% for Sirkana. Finally, the fiber content, determined by acid and alkaline digestion using a Fibertec 2010 automated analyzer, showed 12.25% for Baltimore, 13.44% for Belgrado, 14.23% for Niagara, and 15.23% for Sirkana [12,56]. Baltimore carrot pomace exhibited a total phenolic content (TPC) of 21.45 mg GAE/g and an antioxidant activity (AA) of 19.66%. The Belgrado sample had a TPC of 19.55 mg GAE/g and an AA of 11.10%. The Niagara sample showed a TPC of 20.70 mg GAE/g and an AA of 12.56%, while Sirkana carrot pomace presented a TPC of 24.05 mg GAE/g and an AA of 16.22%.

Wheat flour presented the following chemical properties: moisture content 11.88% (according to ICC methods 110/1 [71]), ash 0.82% (determined by ICC 104/1 method [71]), protein 13.12% (determined by ICC 105/2 protocol [71]), fiber 0.57% (determined by acid and alkaline digestion using a Fibertec 2010 automated analyzer), lipid 1.21% (determined by ICC 104/1 method [71]), wet gluten 12.25% (determined according to ICC 106/1), gluten deformation index 2.50 mm (according to SR 90:2007 [72]), and falling number 592.50 s (determine by ICC 107/1 method [71]).

The mixtures were prepared using a Yucebas Y21 mixer (Izmir, Turkey) by mixing wheat flour with ground carrot pomace for 15 min. To obtain the dough, the flour sample was mixed for 10 min in a laboratory mixer (Kitchen Aid, Whirlpool Corporation, USA) with an amount of water to achieve a dough moisture content of 45%, after which the dough was rested for 15 min before extrusion. The dough was extruded using a Rigatoni pasta die with the Kitchen Aid mixer. The drying of the pasta was carried out in stages, according to the method described by Bergman, Gualberto, and Weber [73], as follows: 30 min in open air at room temperature, then placed in a circulating air oven and dried for 60 min at 40 °C, for 120 min at 80 °C, and for 120 min at 40 °C, then cooled and kept at room temperature in polyethylene bags until the determinations were performed [69].

### 4.2. Rheological Properties of Dough

To examine the viscoelastic behavior of dough during processing, several dynamic testing methods were employed, at least in duplicate, using a Thermo–HAAKE MARS 40 rheometer (Karlsruhe, Germany) with parallel plates at a sample height of 3 mm.

The pasta dough was sheeted to a thickness of 3 mm and rested for 1 h before testing. The dough samples were thermostated and stabilized for 120 s at 20 °C prior to testing. The temperature of the dough samples was kept constant at 20 °C using a Peltier heating system. Excess dough was removed, and to prevent moisture loss during the measurements, a layer of Vaseline was applied to the outer edge of the sample. Before determining the variation in the elastic and viscous moduli with frequency, the limits of the linear viscoelastic region (LVR) of the dough samples were tested by applying a range of stresses from 0.1 to 100 Pa at a constant oscillation frequency of 1 Hz. Consequently, for subsequent rheological tests, a stress of 15 Pa, which falls within the linear viscoelastic region, was selected [74].

The evaluation of the variation in the complex modulus (G*) with frequency was performed in triplicate by applying a constant stress of 15 Pa (within the LVR) and a frequency range from 0.1 to 20 Hz [75].

To assess the behavior of pasta dough upon heating, an oscillatory test was conducted at a constant frequency of 1 Hz, while the temperature was varied from 20 to 100 °C at a rate of 4 °C/min, and the storage modulus (G′) and loss modulus (G″) were recorded.

The viscoelastic behavior of the pasta dough was also evaluated using a creep test, which allowed for the measurement of compliance (J) at a constant temperature of 20 °C. This was performed by suddenly applying a stress of 50 Pa, which was maintained for 60 s and then removed to allow the sample to recover for 180 s.

### 4.3. Dough Texture

To evaluate the texture of the pasta dough, 50 g spheres of dough were formed and then subjected to a double compression down to 50% of their initial height using a Perten TVT–6700 texturometer (Perten Instruments, Sweden) and a cylindrical probe with a diameter of 35 mm. During the test, a speed of 5.0 mm/s, a trigger force of 20 g, and a recovery time of 12 s between compressions were applied. Dough hardness, springiness, gumminess, and resilience were recorded.

### 4.4. Pasta Fracturability

The texture of dry pasta was evaluated by applying a fracturability test using a Perten TVT–6700 texturometer (Perten Instruments, Sweden). For this purpose, a breaking fixture with a sample holding system adjusted to a width of 13 mm was used. Individual pieces of pasta were subjected to a flexural strength test at a testing speed of 3 mm/s and a trigger force of 50 g [74]. Measurements were repeated at least 10 times.

### 4.5. Color Properties of Pasta

The color of the dry pasta was determined according to the EN ISO/CIE 11664-4:2019 [76] standard using a Konica Minolta CR—400 colorimeter (Tokyo, Japan) by reflectance, using the CIE-Lab system (Commission Internationale de l’Eclairage). The color parameters measured were lightness L* (0 = black, 100 = white), a* (intensity of red (+) or green (−) hue), and b* (intensity of yellow (+) or blue (−) hue). Three measurements were taken for each sample. Yellow (YI), white (WI), and browning indexes (BI) were calculated according to previously described methods [77].

### 4.6. Pasta Cooking Behavior

The optimum cooking time (OCT) of the pasta was determined in triplicate according to the method described by Espinosa-Solis et al. [78], following a modified protocol of the AACC Method 66-50. After a cooking time of 4 min, compressions of a piece of pasta were performed between two glass plates every 30 s. OCT was recorded when no more white starch particles were observed after compression. To evaluate the loss of solid substances during cooking (CSL) and water absorption (WA), a quantity of 10 g of pasta was cooked in 20 mL of water according to the OCT, and the resulting liquid was collected and dried at a temperature of 105 °C until a constant mass was achieved. Then, the resulting residue and cooked pasta were weighed. Determinations were performed in triplicate.

### 4.7. Cooked Pasta Texture

The determination of the firmness, cohesiveness, elasticity, resilience, and gumminess of cooked pasta using the Perten TVT-6700 texturometer (Perten Instruments, Sweden) (Figure 6) was performed by applying a double compression of up to 75% of the sample’s initial height, using a cylindrical probe with a diameter of 35 mm, at a speed of 2.0 mm/s, a trigger force of 10 g, and a recovery time of 12 s between compressions. At least 7 measurements were made.

### 4.8. Statistical Processing of Data

The data underwent statistical processing using XLSTAT software for Excel 2024 version (Addinsoft, New York, NY, USA). To determine significant differences between samples, a one-way ANOVA was conducted, followed by Tukey’s post hoc test at a 95% confidence level. Relationships between the measured variables were evaluated through Principal Component Analysis (PCA), and the components were included if the eigenvalue > 1. All measurements were carried out at least in triplicate to ensure reliability.

## Figures and Tables

**Table 1:** Influence of carrot pomace on various food products.

Ingredient Added	Type of Product	Addition Level (%)	Changes Observed	References
Carrot pomace—unspecified variety	Pasta from semolina	10–30	- increased pasta water absorption and cooking loss of solids; - decreased lightness an intensified red nuance, while the yellow nuance decreased; - increased cooked pasta hardness compared to the control; - decreased springiness and chewiness.	Kultys et al. [19]
Carrot pomace—unspecified variety	Wheat pasta with eggs	20	- increased fiber content; - higher color, smell, taste, texture, and general acceptability scores at 20% addition level.	Carrillo [20]
Carrot pomace—unspecified variety	Common wheat pasta	5–25	- decreased cutting force of pasta; - increased viscosity parameters; - enhanced color; - decreased texture.	Mishra and Bhatt [21]
Carrot pomace—unspecified variety	Gluten free muffins	2.5–5	- enhanced fiber content, antioxidant activity, and sensory properties; - decreased lightness, increased red nuance, and decreased yellow tone; - increased muffins hardness and decreased cohesiveness, elasticity, and chewiness.	Oncică et al. [22]
Carrot pomace (Dacus carota)—unspecified variety	Cookies	10–20	- increased flour water absorption capacity; - increased dough water absorption, dough development time, and degree of softening and decreased dough stability and mixing tolerance; - increased G’ and G”; - decreased cookie lightness, increased red and yellow nuances; - decreased hardness.	Ahmad et al. [23]
Carrot pomace—Nanco variety	Cookies	10–25	- increased flour water absorption and decreased dough stability; - decreased lightness and yellow nuance and increased red nuance of cookies; - increased breaking strength; - decreased texture and overall acceptability.	Turksoy and Özkaya [24]
Carrot pomace—unspecified variety	Wheat rolls	1–10	- increased water absorption, dough development time and dough stability, and decreased mixing tolerance index; - decreased loaf volume;	Kohajdová et al. [25]

**Table 2:** Texture parameters of dough.

Sample	Hardness (g)	Springiness (%)	Gumminess (g)	Resilience (adim.)	Cohesiveness (adim.)
**F1**	2849.74 ± 75.71 ^bB^	100.11 ± 0.05 ^aA^	1073.73 ± 14.19 ^bB^	1.04 ± 0.05 ^aA^	0.36 ± 0.01 ^aA^
**F1Ba3**	3227.33 ± 353.43 ^abAB^	99.82 ± 0.28 ^bB^	1137.72 ± 87.31 ^abAB^	0.97 ± 0.08 ^aA^	0.35 ± 0.02 ^aA^
**F1Ba6**	3377.33 ± 175.68 ^abAB^	99.87 ± 0.05 b ^AB^	1232.50 ± 49.69 ^abAB^	1.15 ± 0.04 ^aA^	0.39 ± 0.02 ^aA^
**F1Ba9**	4155.00 ± 100.14 ^abA^	99.75 ± 0.00 ^bB^	1521.32 ± 63.86 ^abA^	1.01 ± 0.06 ^aA^	0.37 ± 0.02 ^aA^
**F1Ba12**	5080.67 ± 402.73 ^abB^	99.63 ± 0.02 ^bB^	1853.42 ± 192.39 ^abB^	0.96 ± 0.03 ^aA^	0.33 ± 0.02 ^aA^
**F1Be3**	3208.08 ± 284.00 ^abAB^	100.14 ± 0.05 ^abB^	1324.19 ± 95.09 ^abAB^	1.12 ± 0.04 ^aA^	0.39 ± 0.02 ^aA^
**F1Be6**	3450.00 ± 348.89 ^abAB^	99.75 ± 0.09 ^abAB^	1354.16 ± 121.41 ^abAB^	1.05 ± 0.04 ^aA^	0.34 ± 0.02 ^aA^
**F1Be9**	3818.00 ± 42.93 ^abA^	99.72 ± 0.00 ^abB^	1389.49 ± 134.07 ^abA^	0.97 ± 0.01 ^aA^	0.33 ± 0.01 ^aA^
**F1Be12**	4663.50 ± 194.50 ^abB^	99.72 ± 0.09 ^abB^	1745.95 ± 67.18 ^abB^	0.87 ± 0.07 ^aA^	0.31 ± 0.02 ^aA^
**F1Ni3**	4077.33 ± 85.58 ^aAB^	99.91 ± 0.01 ^bB^	1327.65 ± 81.91 ^aAB^	1.23 ± 0.02 ^aA^	0.40 ± 0.02 ^aA^
**F1Ni6**	4273.33 ± 113.39 ^aAB^	99.75 ± 0.08 ^bAB^	1560.05 ± 107.04 ^aAB^	1.11 ± 0.04 ^aA^	0.36 ± 0.01 ^aA^
**F1Ni9**	4678.50 ± 425.50 ^aA^	99.73 ± 0.00 ^bB^	1849.72 ± 182.12 ^aA^	1.09 ± 0.06 ^aA^	0.32 ± 0.03 ^aA^
**F1Ni12**	4615.33 ± 381.50 ^aB^	99.67 ± 0.00 ^bB^	1863.02 ± 18.15 ^aB^	0.93 ± 0.03 ^aA^	0.34 ± 0.01 ^aA^
**F1Si3**	2832.67 ± 182.04 ^bAB^	99.83 ± 0.01 ^bB^	1133.29 ± 20.05 ^bAB^	1.18 ± 0.10 ^aA^	0.41 ± 0.02 ^aA^
**F1Si6**	3288.67 ± 87.09 ^bAB^	99.80 ± 0.05 ^bAB^	1331.60 ± 67.07 ^bAB^	1.11 ± 0.08 ^aA^	0.37 ± 0.00 ^aA^
**F1Si9**	4042.00 ± 107.95 ^bA^	99.50 ± 0.00 ^bB^	1485.61 ± 47.11 ^bA^	1.07 ± 0.04 ^aA^	0.36 ± 0.01 ^aA^
**F1Si12**	4298.00 ± 292.18 ^bB^	99.50 ± 0.08 ^bB^	1516.23 ± 77.73 ^bB^	0.95 ± 0.04 ^aA^	0.35 ± 0.02 ^aA^

a–b—different small letters in the same column suggest significant differences (*p* < 0.05) among carrot pomace varieties; A–B—different uppercase letters in the same column suggest significant differences (*p* < 0.05) among addition levels; Ba—Baltimore, Be—Belgrado, Ni—Niagara, Si—Sirkana, F—durum wheat, 3–12—addition levels.

**Table 3:** Color parameters of dry pasta.

Sample	L*	a*	b*	YI	WI	BI
**F1**	71.71 ± 0.39 ^aA^	0.05 ± 0.05 ^cD^	17.64 ± 0.17 ^bC^	35.15 ± 0.50 ^dB^	66.66 ± 0.41 ^aA^	27.62 ± 0.50 ^dB^
**F1Ba3**	77.02 ± 0.08 ^aAB^	1.33 ± 0.05 ^abC^	25.13 ± 0.03 ^aA^	46.60 ± 0.10 ^cA^	65.93 ± 0.08 ^bB^	39.79 ± 0.14 ^cA^
**F1Ba6**	72.20 ± 0.21 ^aABC^	2.09 ± 0.02 ^abB^	24.21 ± 0.06 ^aA^	47.91 ± 0.27 ^cA^	63.07 ± 0.20 ^bBC^	42.02 ± 0.29 ^cA^
**F1Ba9**	69.78 ± 0.96 ^aBC^	2.51 ± 0.02 ^abAB^	21.20 ± 0.26 ^aB^	43.41 ± 0.06 ^cA^	62.99 ± 0.63 ^bBC^	38.09 ± 0.11 ^cA^
**F1Ba12**	66.92 ± 0.12 ^aC^	2.57 ± 0.08 ^abA^	20.70 ± 0.03 ^aB^	44.18 ± 0.03 ^cA^	60.90 ± 0.09 ^bC^	39.04 ± 0.12 ^cA^
**F1Be3**	69.17 ± 0.25 ^bAB^	0.56 ± 0.13 ^bC^	24.46 ± 0.20 ^aA^	50.52 ± 0.23 ^aA^	60.64 ± 0.07 ^dB^	43.07 ± 0.38 ^aA^
**F1Be6**	66.34 ± 0.27 ^bABC^	1.84 ± 0.03 ^bB^	24.31 ± 0.41 ^aA^	52.35 ± 1.04 ^aA^	58.44 ± 0.43 ^dBC^	46.52 ± 1.14 ^aA^
**F1Be9**	65.59 ± 0.48 ^bBC^	1.95 ± 0.05 ^bAB^	23.66 ± 0.03 ^aB^	51.54 ± 0.43 ^aA^	58.19 ± 0.41 ^dBC^	45.82 ± 0.52 ^aA^
**F1Be12**	64.92 ± 0.10 ^bC^	2.07 ± 0.01 ^bA^	21.47 ± 0.01 ^aB^	47.25 ± 0.05 ^aA^	58.82 ± 0.08 ^dC^	41.59 ± 0.05 ^aA^
**F1Ni3**	68.29 ± 0.45 ^bAB^	1.44 ± 0.03 ^abC^	23.63 ± 0.40 ^aA^	49.42 ± 0.65 ^bA^	60.43 ± 0.25 ^dB^	42.95 ± 0.66 ^bA^
**F1Ni6**	67.47 ± 0.32 ^bABC^	2.18 ± 0.02 ^abB^	22.11 ± 0.01 ^aA^	46.82 ± 0.24 ^bA^	60.60 ± 0.27 ^dBC^	41.18 ± 0.26 ^bA^
**F1Ni9**	65.37 ± 0.19 ^bBC^	2.44 ± 0.02 ^abAB^	21.45 ± 0.13 ^aB^	46.88 ± 0.36 ^bA^	59.19 ± 0.20 ^dBC^	41.63 ± 0.39 ^bA^
**F1Ni12**	63.12 ± 0.08 ^bC^	2.52 ± 0.02 ^abA^	21.32 ± 0.09 ^aB^	48.24 ± 0.14 ^bA^	57.33 ± 0.03 ^dC^	43.21 ± 0.16 ^bA^
**F1Si3**	69.30 ± 0.05 ^abAB^	1.89 ± 0.07 ^aC^	23.99 ± 0.31 ^aA^	49.45 ± 0.67 ^bA^	61.00 ± 0.22 ^cB^	43.46 ± 0.60 ^abA^
**F1Si6**	69.18 ± 0.67 ^abABC^	2.31 ± 0.03 ^aB^	23.43 ± 0.48 ^aA^	48.39 ± 1.28 ^bA^	61.21 ± 0.71 ^cBC^	42.85 ± 1.29 ^abA^
**F1Si9**	68.64 ± 0.24 ^abBC^	2.70 ± 0.05 ^aAB^	22.71 ± 0.21 ^aB^	47.26 ± 0.26 ^bA^	61.19 ± 0.07 ^cBC^	42.17 ± 0.31 ^abA^
**F1Si12**	67.83 ± 0.43 ^abC^	2.85 ± 0.03 ^aA^	22.59 ± 0.11 ^aB^	47.58 ± 0.08 ^bA^	60.59 ± 0.29 ^cC^	42.70 ± 0.07 ^abA^

a–d—different small letters in the same column suggest significant differences (*p* < 0.05) among carrot pomace varieties; A–D—different uppercase letters in the same column suggest significant differences (*p* < 0.05) among addition levels; Ba—Baltimore, Be—Belgrado, Ni—Niagara, Si—Sirkana, F—durum wheat, 3–12—addition levels, L*—lightness, a*—red-green nuance, b*—blue-yellow nuance, YI—yellow index, WI—white index, BI—browning index.

**Table 4:** Texture parameters of cooked pasta.

Sample	Elasticity (%)	Chewiness (g)	Gumminess (g)	Cohesiveness (Adim.)	Hardness (g)	Adhesiveness (J)	Resilience (Adim.)	Stickiness (g)
**F1**	99.72 ± 0.21 ^bB^	1071.81 ± 15.38 ^cC^	1073.67 ± 14.13 ^cC^	0.36 ±0.01 ^aC^	2818.40 ± 239.46 ^bB^	−90.49 ± 9.50 ^abA^	1.02 ± 0.06 ^cD^	−49.99 ± 3.83 ^aA^
**F1Ba3**	99.79 ± 0.08 ^bAB^	2688.88 ± 219.26 ^abA^	2693.38 ± 220.32 ^abA^	0.68 ± 0.02 ^bB^	3243.22 ± 299.16 ^aB^	−10.51 ± 0.29 ^bA^	7.10 ± 0.53 ^abA^	−74.98 ± 3.27 ^bA^
**F1Ba6**	99.79 ± 0.09 ^bAB^	2518.87 ± 240.34 ^abAB^	2527.85 ± 242.78 ^abAB^	0.71 ± 0.04 ^bAB^	3361.24 ± 353.80 ^aAB^	−84.14 ± 3.40 ^bA^	6.52 ± 0.61 ^abB^	−154.37 ± 3.44 ^bA^
**F1Ba9**	99.81 ± 0.06 ^bAB^	2402.90 ± 195.15 ^abAB^	2408.77 ± 196.10 ^abAB^	0.71 ± 0.05 ^bAB^	3491.71 ± 349.13 ^aA^	−124.68 ± 4.47 ^bA^	6.28 ± 0.59 ^abBC^	−208.81 ± 19.65 ^bA^
**F1Ba12**	99.84 ± 0.01 ^bA^	2059.64 ± 134.58 ^abB^	2063.28 ± 133.84 ^abB^	0.71 ± 0.01 ^bA^	3788.88 ± 229.31 ^aA^	−187.11 ± 19.21 ^bA^	5.94 ± 0.46 ^abC^	−208.74 ± 23.14 ^bA^
**F1Be3**	99.81 ± 0.08 ^abAB^	2953.62 ± 220.82 ^aA^	2959.00 ± 221.66 ^abA^	0.71 ± 0.05 ^aB^	3305.99 ± 317.24 ^aB^	−136.22 ± 2.45 ^cA^	7.28 ± 0.66 ^bA^	−119.92 ± 13.38 ^cA^
**F1Be6**	99.85 ± 0.01 ^abAB^	2941.82 ± 201.87 ^aAB^	2949.08 ± 206.00 ^aAB^	0.73 ± 0.03 ^aAB^	3347.85 ± 383.60 ^aAB^	−239.84 ± 9.35 ^cA^	6.20 ± 0.47 ^bB^	−224.45 ± 6.00 ^cA^
**F1Be9**	99.84 ± 0.01 ^abAB^	2658.44 ± 249.31 ^aAB^	2665.22 ± 249.45 ^aAB^	0.76 ± 0.04 ^aAB^	3547.67 ± 390.04 ^aA^	−270.34 ± 24.25 ^cA^	5.95 ± 0.48 ^bBC^	−330.78 ± 19.11 ^cA^
**F1Be12**	99.92 ± 0.12 ^abA^	2041.54 ± 219.18 ^aB^	2045.39 ± 217.89 ^aB^	0.77 ± 0.02 ^aA^	3686.12 ± 262.54 ^aA^	−237.00 ± 21.82 ^cA^	4.82 ± 0.24 ^bC^	−345.46 ± 32.32 ^cA^
**F1Ni3**	99.85 ± 0.01 ^aAB^	2268.82 ± 180.14 ^bA^	2273.12 ± 181.47 ^aA^	0.68 ± 0.03 ^abB^	2417.29 ± 189.16 ^bB^	−23.17 ± 2.22 ^aA^	7.94 ± 0.54 ^aA^	−11.20 ± 1.76 ^aA^
**F1Ni6**	99.95 ± 0.08 ^aAB^	2034.08 ± 189.65 ^bAB^	2037.94 ± 190.92 ^bAB^	0.69 ± 0.01 ^abAB^	3124.16 ± 265.67 ^bAB^	−12.15 ± 0.35 ^aA^	7.31 ± 0.56 ^aB^	−56.58 ± 8.69 ^aA^
**F1Ni9**	99.98 ± 0.15 ^aAB^	1984.24 ± 88.57 ^bAB^	1989.82 ± 91.49 ^bAB^	0.72 ± 0.05 ^abAB^	3318.97 ± 324.67 ^bA^	−16.39 ± 6.52 ^aA^	6.83 ± 0.54 ^aBC^	−52.08 ± 1.69 ^aA^
**F1Ni12**	100.04 ± 0.24 ^aA^	2254.41 ± 213.28 ^bB^	2257.66 ± 211.07 ^bB^	0.74 ± 0.02 ^abA^	3399.38 ± 391.15 ^bA^	−33.22 ± 6.70 ^aA^	6.49 ± 0.40 ^aC^	−89.20 ± 5.67 ^aA^
**F1Si3**	99.76 ± 0.09 ^abAB^	2379.45 ± 198.14 ^bA^	2385.16 ± 198.66 ^bA^	0.68 ± 0.04 ^bB^	3183.68 ± 118.07 ^abB^	−54.13 ± 7.21 ^abA^	8.14 ± 0.76 ^aA^	−142.03 ± 13.00 ^abA^
**F1Si6**	99.82 ± 0.07 ^abAB^	2247.78 ± 234.60 ^bAB^	2254.05 ± 235.93 ^bAB^	0.70 ± 0.04 ^bAB^	3212.93 ± 195.00 ^abAB^	−73.42 ± 1.12 ^abC^	6.89 ± 0.66 ^aB^	−165.24 ± 18.15 ^ab^
**F1Si9**	99.88 ± 0.07 ^abAB^	2226.47 ± 39.14 ^bAB^	2231.91 ± 40.63 ^bAB^	0.71 ± 0.02 ^bAB^	3317.31 ± 213.20 ^abA^	−62.79 ± 0.73 ^abC^	6.68 ± 0.46 ^aBC^	−124.25 ± 13.30 ^ab^
**F1Si12**	99.92 ± 0.09 ^abA^	2084.92 ± 100.92 ^bB^	2088.93 ± 99.76 ^bB^	0.70 ± 0.00 ^bA^	3420.43 ± 119.65 ^abA^	−29.83 ± 5.58 ^abC^	6.52 ± 0.34 ^aC^	−65.96 ± 3.89 ^ab^

a–c—different small letters in the same column suggest significant differences (*p* < 0.05) among carrot pomace varieties; A–D—different uppercase letters in the same column suggest significant differences (*p* < 0.05) among addition levels; Ba—Baltimore, Be—Belgrado, Ni—Niagara, Si—Sirkana, F—durum wheat, 3–12—addition levels.

**Table 5:** Eigenvalue factors.

	PC1	PC2	PC3	PC4	PC5
**Eigenvalue**	10.19	6.31	3.57	2.04	1.19
**Variability (%)**	39.19	24.28	13.75	7.84	4.57
**Cumulative %**	39.19	63.47	77.21	85.05	89.62

PC—principal component.

**Table 6:** Contribution of the variables (%).

Variable	PC1	PC2	PC3	PC4	PC5
**Dough hardness**	7.24	1.02	1.75	0.75	4.48
**Dough springiness**	6.04	0.00	0.87	3.79	0.19
**Dough gumminess**	7.47	0.82	0.86	3.22	3.06
**Dough resilience**	3.13	3.11	5.97	1.04	1.01
**Dough cohesiveness**	4.21	3.56	2.31	0.24	2.90
G*_10Hz_	4.70	1.35	0.87	6.64	12.46
**J_max_**	6.05	1.83	0.34	0.96	13.22
**T_i_**	2.81	0.44	1.10	16.74	13.74
**T_g_**	5.37	0.05	3.90	7.73	6.00
L*	4.88	0.01	0.00	21.32	0.42
a*	6.37	0.20	1.96	2.65	2.07
b*	0.02	14.01	0.07	3.22	1.89
**YI**	1.16	12.83	0.13	0.86	3.48
**WI**	5.02	2.22	0.03	14.74	1.62
**BI**	2.30	11.23	0.01	0.49	2.42
**WA**	0.00	2.93	14.23	2.01	11.05
**CSL**	7.50	1.07	0.16	0.34	0.70
**Pasta fracturability**	6.80	0.23	2.17	0.25	6.19
**Pasta elasticity**	5.93	0.00	4.73	4.98	3.48
**Pasta chewiness**	0.19	12.42	2.47	1.33	0.00
**Pasta gumminess**	0.19	12.42	2.47	1.34	0.00
**Pasta cohesiveness**	5.25	6.65	0.10	0.06	1.05
**Pasta firmness**	4.67	0.03	7.31	3.60	2.87
**Pasta adhesiveness**	0.69	0.01	22.15	1.38	0.29
**Pasta resilience**	0.44	11.39	4.65	0.27	3.24
**Pasta stickiness**	1.59	0.16	19.38	0.04	2.16

PC—principal component, G*_10 Hz_—complex modulus at 10 Hz frequency, J_max_—maximum compliance, T_i_—initial gelatinization temperature, T_g_—glass transition temperature, L*—lightness, a*—red-green nuance, b*—blue-yellow nuance, YI—yellow index, WI—white index, BI—browning index, WA—pasta water absorption, CSL—cooking solids loss.

**Table 7:** Contribution of the samples (%).

Sample	PC1	PC2	PC3	PC4	PC5
**F1**	33.09	51.97	1.48	1.88	3.27
**F1Ba3**	6.44	1.61	0.07	21.63	0.20
**F1Ba6**	0.58	1.56	0.03	17.29	0.02
**F1Ba9**	0.39	1.18	0.96	5.58	25.66
**F1Ba12**	6.53	5.14	1.29	0.36	30.76
**F1Be3**	4.12	8.77	3.35	1.73	0.26
**F1Be6**	0.20	6.95	9.99	1.28	5.95
**F1Be9**	2.36	2.00	20.92	1.65	3.09
**F1Be12**	9.89	3.58	11.31	2.44	0.03
**F1Ni3**	3.88	4.19	16.42	7.40	0.41
**F1Ni6**	0.03	0.00	11.67	2.27	1.76
**F1Ni9**	5.01	1.08	13.25	2.04	1.61
**F1Ni12**	8.41	0.47	2.01	12.73	0.39
**F1Si3**	9.37	8.84	0.21	1.69	7.29
**F1Si6**	1.49	1.10	0.06	0.19	0.52
**F1Si9**	1.07	0.03	1.82	3.15	2.78
**F1Si12**	7.15	1.52	5.15	16.69	16.01

PC—principal component, Ba—Baltimore, Be—Belgrado, Ni—Niagara, Si—Sirkana, F—durum wheat, 3–12—addition levels.

## Data Availability

Data will be available upon request.

## Supplementary Materials

The following supporting information can be downloaded at: https://www.mdpi.com/article/10.3390/gels11070481/s1, Table S1: Correlations between variables.
