# Supplementary material for: Physical Characteristics of Durum Wheat Dough and Pasta with Different Carrot Pomace Varieties
Source: Gels. 2025 Jun 22;11(7):481. doi: 10.3390/gels11070481 (PMC12294662; doi:10.3390/gels11070481)
Supplement: Supplementary file 1 [file gels-11-00481-s001.zip › gels-3692863-supplementary.pdf]

Table S1. Correlations between variables

Variables	Dough hardness	Dough springiness	Dough gumminess	Dough resilience	Dough cohesiveness	G^*_{10Hz}	J_{max}	T_i	T_g	L*	a*	b*	YI	WI	BI	WA	CSL	Pasta fracturability	Pasta elasticity	Pasta chewiness	Pasta gumminess	Pasta cohesiveness	Pasta firmness	Pasta adhesiveness	Pasta resilience	Pasta stickiness
Dough hardness	**1**																									
Dough springiness	**-0.64**	**1**																								
Dough gumminess	**0.94**	**-0.57**	**1**																							
Dough resilience	-0.46	0.44	-0.47	**1**																						
Dough cohesiveness	**-0.62**	0.47	**-0.65**	**0.80**	**1**																					
G^*_{10Hz}	**0.79**	**-0.62**	**0.66**	-0.41	-0.37	**1**																				
J_{max}	**-0.75**	**0.56**	**-0.75**	**0.55**	**0.70**	**-0.51**	**1**																			
T_i	0.38	**-0.55**	0.30	-0.29	-0.39	**0.51**	**-0.56**	**1**																		
T_g	**0.67**	**-0.68**	**0.58**	-0.31	-0.41	**0.70**	**-0.72**	**0.86**	**1**																	
L*	**-0.64**	0.40	**-0.75**	0.26	0.46	-0.26	**0.67**	-0.06	-0.28	**1**																
a*	**0.66**	**-0.90**	**0.64**	-0.25	-0.32	**0.67**	-0.48	**0.52**	**0.65**	-0.44	**1**															
b*	-0.33	-0.01	-0.35	0.33	0.41	-0.30	0.34	0.01	0.04	0.25	0.07	**1**														
YI	0.02	-0.23	0.07	0.18	0.14	-0.16	-0.04	0.04	0.19	-0.33	0.30	**0.84**	**1**													
WI	**-0.53**	0.42	**-0.63**	0.15	0.31	-0.14	**0.55**	-0.07	-0.32	**0.90**	**-0.49**	-0.19	**-0.69**	**1**												
BI	0.16	-0.39	0.20	0.10	0.05	-0.02	-0.15	0.13	0.30	-0.41	0.47	**0.77**	**0.98**	**-0.76**	**1**											
WA	0.22	-0.01	0.16	**0.57**	0.33	0.04	0.20	-0.13	0.11	-0.05	0.20	0.29	0.30	-0.18	0.30	**1**										
CSL	**0.78**	**-0.74**	**0.83**	**-0.54**	**-0.54**	**0.72**	**-0.71**	**0.54**	**0.64**	**-0.60**	**0.81**	-0.30	0.03	**-0.50**	0.20	-0.13	**1**									
Pasta fracturability	**0.78**	-0.46	**0.75**	**-0.64**	**-0.64**	**0.76**	**-0.60**	0.39	**0.55**	**-0.51**	**0.52**	-0.15	0.13	-0.47	0.23	-0.10	**0.69**	**1**								
Pasta elasticity	**0.76**	**-0.56**	**0.83**	-0.33	**-0.50**	0.40	**-0.79**	0.34	**0.65**	**-0.72**	**0.59**	-0.11	0.29	**-0.71**	0.40	0.29	**0.65**	0.48	**1**							
Pasta chewiness	-0.18	-0.02	-0.14	0.11	0.24	-0.11	0.20	-0.08	0.03	0.06	0.10	**0.88**	**0.82**	-0.33	**0.77**	0.12	-0.16	0.15	-0.04	**1**						
Pasta gumminess	-0.18	-0.02	-0.14	0.11	0.24	-0.11	0.20	-0.08	0.03	0.06	0.10	**0.88**	**0.82**	-0.33	**0.77**	0.12	-0.16	0.15	-0.04	**0.99**	**1**					
Pasta cohesiveness	0.48	**-0.53**	**0.50**	-0.16	-0.18	0.34	-0.31	0.25	0.46	-0.44	**0.66**	**0.58**	**0.81**	**-0.71**	**0.87**	0.29	**0.49**	**0.58**	**0.51**	**0.69**	**0.69**	**1**				
Pasta firmness	0.43	**-0.51**	**0.53**	**-0.68**	**-0.58**	**0.49**	-0.33	0.35	0.35	-0.31	**0.52**	-0.01	0.15	-0.32	0.24	-0.37	**0.68**	**0.74**	0.24	0.25	0.25	**0.53**	**1**			
Pasta adhesiveness	-0.07	0.00	-0.10	0.41	0.40	-0.04	0.11	0.09	0.16	0.34	0.06	0.01	-0.20	0.33	-0.18	**0.54**	-0.11	**-0.50**	0.19	-0.27	-0.27	-0.24	**-0.53**	**1**		
Pasta resilience	0.10	-0.26	0.09	0.41	0.41	0.04	0.18	0.00	0.21	-0.05	0.43	**0.76**	**0.76**	-0.38	**0.77**	**0.71**	0.03	0.00	0.26	**0.66**	**0.66**	**0.71**	-0.02	0.32	**1**	
Pasta stickiness	-0.13	0.20	-0.17	0.48	0.41	-0.12	0.11	-0.01	0.09	0.31	-0.21	-0.09	-0.27	0.36	-0.30	0.46	-0.30	**-0.57**	0.09	-0.31	-0.31	-0.45	**-0.69**	**0.89**	0.15	**1**

Values in bold are different from 0 with a significance level alpha = 0.05, G^*_{10Hz} - complex modulus at 10 Hz frequency, J_{max} – maximum compliance, T_i – initial gelatinization temperature, T_g – glass transition temperature, L* - lightness, a* - red-green nuance, b* - blue-yellow nuance, YI – yellow index, WI – white index, BI – browning index, WA – pasta water absorption, CSL – cooking solids loss.
